# The Independent and Combined Associations of Multimorbidity and Polypharmacy on Periodontal Disease Severity and Progression in Older Adults

**DOI:** 10.4317/jced.64297

**Published:** 2026-07-29

**Authors:** Georgios S. Chatzopoulos, Larry F. Wolff

**Affiliations:** 1 DDS, MS, Department of Developmental and Surgical Sciences, Division of Periodontology, School of Dentistry, University of Minnesota, 515 Delaware Street SE, Minneapolis, MN,55455, USA & Department of Preventive Dentistry, Periodontology and Implant Biology, School of Dentistry, Aristotle University of Thessaloniki, 54124 Thessaloniki, Greece; 2 DDS, MS, PhD, Department of Developmental and Surgical Sciences, Division of Periodontology, School of Dentistry, University of Minnesota, 515 Delaware Street SE, Minneapolis, MN,55455, USA

## Abstract

**Background:**

The aging global population is characterized by a high prevalence of multimorbidity and polypharmacy. This study aimed to investigate the independent and combined associations of these factors on the severity and progression of periodontal disease in older adults and to develop a predictive machine learning model for clinically meaningful disease progression.

**Materials and Methods:**

This retrospective cohort study utilized de-identified electronic health records from patients aged 65 and older with periodontitis. The primary independent variables were polypharmacy (categorized as 0-4, 5-9, or 10+ medications) and a multimorbidity index (a sum of self-reported systemic conditions). The outcome variables were baseline mean clinical attachment level (MEAN_CAL) and the annualized rate of MEAN_CAL progression. Multivariable linear regression models were used to assess associations, and a Random Forest Classifier was developed to predict clinically relevant disease progression (CAL 1mm).

**Results:**

The study included 5,645 patients for cross-sectional analysis and a sub-cohort of 2,130 for longitudinal analysis. Both polypharmacy (5-9 medications: 0.18 mm; 10+ medications: 0.29 mm) and each additional systemic disease (0.09 mm) were significantly associated with higher baseline MEAN_CAL (p < 0.001). Longitudinally, hyperpolypharmacy (10+ medications) and each additional comorbidity were associated with an annual CAL loss of 0.08 mm (p = 0.002) and 0.03 mm (p = 0.001), respectively. The machine learning model predicted disease progression with an AUC-ROC of 0.81, 94% specificity, and 61% recall. Baseline MEAN_CAL, smoking, age, bleeding on probing, diabetes, and the number of medications were the most important predictors.

**Conclusions:**

Multimorbidity and polypharmacy are significant and clinically relevant independent predictors of more severe periodontal disease and faster progression in older adults. Predictive modeling can help identify high-risk individuals, emphasizing the need for integrated medical-dental care for this population.

## Introduction

The global population is undergoing a significant demographic shift, with the proportion of older adults increasing at an unprecedented rate (1). This aging trend is accompanied by a higher prevalence of chronic systemic diseases, leading to a state of multimorbidity-the coexistence of two or more chronic conditions-becoming the norm rather than the exception in geriatric populations (2 , 3). Consequently, the management of these multiple conditions often necessitates complex therapeutic regimens, resulting in polypharmacy, commonly defined as the concurrent use of five or more medications (4). While essential for managing systemic health, the cumulative burden of both multiple diseases and multiple medications can have wide-ranging and often unforeseen consequences on other aspects of health, including oral health (5). Periodontal disease, a chronic inflammatory condition leading to the destruction of tooth-supporting tissues, is also highly prevalent among older adults and shares many common risk factors with other chronic diseases (6 , 7). A substantial body of evidence has established a bidirectional relationship between periodontitis and systemic conditions such as diabetes mellitus and cardiovascular disease, largely mediated by shared inflammatory pathways (8 , 9). The systemic inflammatory burden from multimorbidity may therefore predispose older adults to more severe periodontal disease or a more rapid progression of existing disease (10). This complex interplay highlights the need to view periodontal health not in isolation, but as an integral component of an older individual's overall health status. Compounding the effects of multimorbidity is the direct and indirect impact of polypharmacy on the oral cavity. Numerous medications prescribed for common geriatric conditions have well-documented adverse effects on periodontal tissues (11 , 12). One of the most pervasive side effects is xerostomia, or drug-induced dry mouth, which is associated with over 400 commonly used drugs, including antihypertensives, antidepressants, and diuretics (13 , 14). Reduced salivary flow impairs the natural cleansing and antimicrobial functions within the oral cavity, leading to increased plaque biofilm accumulation and a heightened risk for both dental caries and the progression of periodontal disease (15). Beyond xerostomia, other medications can directly alter the host's inflammatory response or cause tissue changes such as drug-induced gingival enlargement, further complicating periodontal health management (11 , 12). While the individual effects of specific diseases or medication classes on periodontal health have been studied, a significant gap exists in the literature regarding the combined, cumulative impact of multimorbidity and polypharmacy. Most studies focus on a single condition, such as the link between diabetes and periodontitis (8), or a single class of drugs (11). However, the typical geriatric patient presents with a complex interplay of multiple diseases and medications, and the synergistic effect of this combined burden on periodontal stability is not well understood (5 , 16). Understanding this relationship is critical for developing comprehensive risk assessment models and integrated care pathways for the aging population. Understanding how the concurrent burdens of multimorbidity and polypharmacy interact over time is essential for comprehensive geriatric care. A significant gap remains regarding the synergistic, longitudinal impact of these combined factors on periodontal health. This study addresses this by not only evaluating the combined effects of systemic disease and medication burden on longitudinal periodontal disease progression, but also by pioneering the application of a predictive machine learning algorithm. By utilizing an advanced, data-driven prognostic model, this research offers a novel approach for identifying high-risk older adults and accurately anticipating future clinically meaningful attachment loss. Therefore, the aim of this study was to investigate the independent and combined associations of multimorbidity and polypharmacy on both the baseline severity and the longitudinal progression of periodontal disease in a large cohort of older adults. In addition, the aim was to develop and validate a machine learning model to predict clinically meaningful periodontal disease progression in a large, real-world cohort of older adults. It was hypothesized that a higher number of systemic comorbidities (multimorbidity) and a greater number of prescribed medications (polypharmacy) would be independently associated with more severe baseline clinical attachment loss. Furthermore, it was hypothesized that these factors would also predict a faster rate of periodontal disease progression over time, even after controlling for established risk factors such as age, gender, and smoking status.

## Materials and Methods

- Study Design and Data Collection This retrospective cohort study was determined by the University of Minnesota Institutional Review Board (STUDY00016576) not to constitute research involving human subjects, as it involved the analysis of a pre-existing, de-identified dataset. The study was conducted in accordance with the principles of the Helsinki Declaration of 1975, as revised in 2013. - Data Source and Patient Selection Electronic health records (EHRs) were extracted from a large dental data repository containing records from patients treated at multiple university dental clinics. The dataset included dental charts of adult patients who had sought dental therapy and consented to the use of their de-identified data for research. All EHR data were entered by dental students, residents, and faculty during routine patient care. The primary cohort consisted of all patients from the periodontitis group aged 65 years or older. Patients were confirmed as having periodontitis if their records contained relevant diagnostic information or specific Current Dental Terminology (CDT) codes for periodontal treatment. A sub-cohort for the longitudinal analysis included patients from the geriatric cohort with at least two periodontal examinations separated by a minimum of two years. - Data Extraction and Variables Data extracted from EHRs included demographics, medication lists, self-reported medical conditions, and periodontal examination records. For patients with multiple examinations, only the initial assessment was used for the baseline cross-sectional analysis. The primary independent variables were: Polypharmacy: The total number of unique medications was counted and categorized as "No Polypharmacy" (0-4 medications), "Polypharmacy" (5-9 medications), and "Hyperpolypharmacy" (10 or more medications). Multimorbidity Index: A numerical index was created by summing the "YES" responses to a list of self-reported systemic conditions, including diabetes, high blood pressure, cardiovascular problems, arthritis, osteoporosis, kidney disease, and cancer. Other independent variables included age, gender, race, ethnicity, and current smoking status. The outcome variables were: Presence/Severity Analysis: The Mean Clinical Attachment Level (MEAN_CAL) in millimeters from the initial periodontal examination. Progression Analysis: The annualized rate of MEAN_CAL progression, calculated as the total change in MEAN_CAL between the first and last examinations divided by the follow-up time in years. Predictive Model: Clinically relevant periodontal disease progression, a binary outcome defined as a change in MEAN CAL of at least 1mm (CAL1mm) between the first and last examinations. Secondary Exploratory Analyses To further delineate the specific biological and pharmacological predictors of periodontal disease, secondary variables were constructed for sub-analyses: Biological Disease Clusters: Rather than relying solely on a simple additive index, the self-reported systemic conditions were categorized into severity-based biological clusters to assess differential impacts. These clusters included Major Cardiovascular (e.g., history of heart attack, stroke, coronary heart disease), Minor Cardiovascular (e.g., hypertension), Metabolic/Endocrine (e.g., diabetes, osteoporosis), Immunological (e.g., arthritis, lupus), and Psychiatric/Neurologic conditions. Xerostomic Medication Burden: To evaluate the specific mechanism of drug-induced hyposalivation, raw patient medication logs were subjected to text-mining. The data was scanned for over 30 specific active pharmacological ingredients with well-documented, severe xerostomic side effects (including specific anticholinergics, SSRIs, and diuretics) to calculate an individualized 'Xerostomic Medication Count', distinct from the broad polypharmacy metric. - Statistical Analysis The statistical analysis began with a descriptive assessment of the geriatric cohort, presenting continuous variables as means and standard deviations (SD) and categorical variables as frequencies and percentages. To assess the independent association of polypharmacy and multimorbidity with periodontal disease, two separate multivariable linear regression models were constructed. The first model evaluated the cross-sectional association between the predictor variables and baseline "MEAN CAL". The second model evaluated the longitudinal association between the baseline predictor variables and the annualized rate of "MEAN CAL" progression. Both models included the Polypharmacy category, the Comorbidity Index, baseline age, gender, and smoking status as predictors. For the primary predictive analysis, the dataset was randomly partitioned into a training set (75% of the cohort) and a testing set (25%). A Random Forest Classifier, a machine learning algorithm well-suited for handling numerous predictors and complex interactions, was trained on the training data. To ensure the robustness of the Random Forest Classifier and to mitigate the risk of overfitting, several methodological steps were rigorously applied. Missing data within the predictor variables were addressed using median imputation for continuous variables and mode imputation for categorical variables. To manage the inherent class imbalance between the stable patients and the smaller subset of patients experiencing clinically meaningful progression, class weights were balanced during the model training phase. The validation strategy employed a stratified k-fold cross-validation approach (k=5) on the training set prior to final evaluation on the unseen testing set. Furthermore, hyperparameters, including the maximum depth of the trees and the number of estimators, were optimized using a grid search methodology to maximize the model's generalizability and calibration. The model's predictive performance was then evaluated on the unseen testing data using several key metrics, including the Area Under the Receiver Operating Characteristic Curve (AUC-ROC), accuracy, precision, recall (sensitivity), and specificity. The relative importance of each baseline predictor in the model was determined using feature importance scores. To complement this, a multivariable logistic regression analysis was performed to quantify the independent association of each risk factor with the outcome, with results presented as Odds Ratios (ORs) and their 95% confidence intervals. The results of the regression models were presented as coefficients with their corresponding 95% confidence intervals. Finally, to explore the granular predictors of baseline periodontal destruction, secondary multivariable linear regression models were constructed by substituting the general polypharmacy and comorbidity indices with the specific biological disease clusters and the xerostomic medication count. For all inferential statistical tests, a p-value of less than 0.05 was considered statistically significant.

## Results

The initial geriatric cohort identified for the cross-sectional analysis of disease severity consisted of 5,645 patients aged 65 and older with complete baseline data. From this group, a sub-cohort of 2,130 patients who had at least two years of follow-up data was established for the longitudinal analysis of disease progression. A comparison of these two groups is presented in Table 1. As expected, the follow-up time was significantly longer for the sub-cohort selected for the progression analysis (p < 0.001). Importantly, for all other key baseline characteristics-including age, gender distribution, racial composition, and initial periodontal severity-there were no statistically significant differences between the larger geriatric cohort and the sub-cohort used for the longitudinal analysis (Table 1).

Table 1

This indicates that the sub-cohort is a representative sample of the larger group, strengthening the validity of the progression analysis findings. The multivariable linear regression analysis for baseline periodontal severity revealed that both polypharmacy and multimorbidity were significantly associated with a higher initial MEAN CAL (Table 2).

Table 2

After adjusting for baseline age, gender, and smoking status, patients with polypharmacy (5-9 medications) and hyperpolypharmacy (10+ medications) had a significantly higher baseline CAL by 0.18 mm and 0.29 mm, respectively, compared to those taking fewer than five medications (p < 0.001 for both). Furthermore, each additional systemic disease in the Comorbidity Index was independently associated with a 0.09 mm increase in baseline CAL (p < 0.001). Smoking status, increasing age, and male gender were also confirmed as significant predictors of more severe baseline disease (Table 2). The longitudinal analysis, which assessed the annualized rate of CAL progression in the sub-cohort of 2,130 patients, identified a similar set of predictors for disease advancement over time (Table 3).

Table 3

Hyperpolypharmacy was a significant predictor, associated with an additional 0.08 mm of CAL loss per year compared to the no-polypharmacy group (p = 0.002). The Comorbidity Index was also a significant predictor, with each additional systemic disease contributing to an extra 0.03 mm of CAL loss per year (p = 0.001). Consistent with the baseline analysis, smoking, increasing age, and male gender were also found to be significant independent predictors for a faster rate of periodontal disease progression (Table 3). To further explore the prediction of disease progression, a machine learning model was developed on a separate cohort of 4,533 patients to predict a worsening of CAL by 1mm or more. The performance of this Random Forest model is detailed in Table 4.

Table 4

The model demonstrated a strong ability to distinguish between groups, achieving an Area Under the Curve (AUC-ROC) of 0.81. The model's clinical utility was highlighted by its high specificity (94%) and a recall (sensitivity) of 61%, indicating it successfully identified a majority of the patients who truly experienced disease progression. The machine learning model also identified the most influential baseline factors for predicting future progression. A patient's existing level of periodontal destruction, Baseline Mean CAL, was overwhelmingly the most powerful predictor. This was followed in importance by Smoking Status, Baseline Age, Baseline Bleeding on Probing (BOP), Diabetes Status, and the Number of Medications (Polypharmacy). These findings from both the regression and machine learning analyses consistently highlight that a patient's clinical, systemic, and behavioral status at baseline are critical determinants of future periodontal health outcomes. - Secondary Exploratory Analyses: Disease Severity and Pharmacological Profiles To address the potential varying impact of specific systemic conditions, a secondary multivariable linear regression analysis was performed where the simple additive comorbidity count was replaced with severity-based biological categories. Major cardiovascular conditions (e.g., history of heart attack, stroke, coronary heart disease) were found to be the strongest systemic predictors of baseline periodontal destruction, independently associated with an increase in MEAN_CAL by 0.20 mm (p < 0.001). Minor cardiovascular conditions similarly increased MEAN_CAL by 0.14 mm (p < 0.001). Conversely, the presence of systemic immunological conditions (e.g., arthritis, lupus) demonstrated a statistically significant negative association with MEAN_CAL (-0.11 mm, p < 0.001) (Table 5).

Table 5

Furthermore, to delineate the pharmacological drivers of periodontal decline, a sub-analysis was conducted isolating the cumulative burden of medications with known xerostomic effects. Text-mining of patient medication logs identified over 30 specific xerostomia-inducing active ingredients (e.g., anticholinergics, SSRIs, diuretics). Interestingly, when replacing the general polypharmacy count with a specific 'Xerostomic Medication Count' in the regression model, the robust destructive association previously observed with broad polypharmacy was not mirrored, yielding a slight negative coefficient (-0.04 mm, p < 0.001).

## Discussion

This study was designed to address a critical issue in geriatric health: the combined association of multimorbidity and polypharmacy on periodontal disease (4). The primary aims were to determine if these factors were associated with disease severity and progression, and to develop a machine learning model to predict significant and clinically meaningful clinical attachment loss in older adults, a population that is rapidly growing worldwide. The findings of this study largely confirmed the initial hypotheses. Both a higher number of systemic comorbidities and a greater number of medications were independently associated with more severe baseline periodontal disease and a faster rate of disease progression, even after controlling for established risk factors. Furthermore, the Random Forest model successfully predicted future progression with good discriminatory power, highlighting the potential of machine learning in geriatric dental care. The results of this study are consistent with and expand upon the existing literature. The identification of smoking and diabetes as significant risk factors for both disease presence and progression aligns with numerous clinical studies and reviews (11 , 12). This study's novel contribution is the quantification of the cumulative burden of multimorbidity and polypharmacy. While previous work has often focused on the correlation of a single disease or medication class, our findings demonstrate that the cumulative number of concurrent conditions and prescriptions are, in themselves, significant risk indicators. This supports the concept that a high systemic disease burden may contribute to a pro-inflammatory state that exacerbates periodontal destruction, a known concern in medically complex patients (4 , 5). The significant association between polypharmacy and adverse periodontal outcomes is a particularly important finding. The mechanisms are likely multifaceted, with drug-induced xerostomia being a primary pathway (12). Many medications commonly taken by older adults, including antihypertensives and antidepressants, are known to cause dry mouth, which impairs the oral cavity's natural cleansing mechanisms and may contribute to increased plaque accumulation and a heightened risk for both dental caries and periodontal disease (4 , 11). These results are in line with previous research that has investigated the relationship between the number of medications taken by the elders and its effect on clinical attachment level, which also identified a need for more comprehensive research (16). While our Random Forest model demonstrated strong overall discriminatory ability (AUC-ROC of 0.81), it is important to contextualize the specific performance metrics of 94% specificity and 61% recall within a clinical framework. A recall of 61% at the default classification threshold means that a proportion of patients who will truly experience disease progression may not be flagged by the initial model. However, a primary clinical advantage of algorithmic risk assessment is the adaptability of the decision threshold. In a real-world, preventive dental setting-where the clinical 'cost' of a false positive (e.g., providing additional oral hygiene instruction or scheduling a more frequent recall visit) is minimal compared to the cost of missed disease progression-this threshold can be intentionally lowered. Adjusting the model to prioritize a higher recall would capture a greater number of at-risk older adults for early intervention, deliberately trading some specificity to align with the ultimate goal of disease prevention. It is important to contextualize the statistical findings of this study within a framework of clinical relevance. Given the large sample size of our primary cohort (N=5,645), even minute numerical differences-such as a 0.18 mm difference in baseline CAL or an accelerated progression rate of 0.08 mm/year-yielded high statistical significance (p < 0.001). While a progression rate of 0.08 mm/year may appear clinically negligible when viewed in isolation over a single year, its cumulative impact is substantial. Over the course of a decade, this rate translates to nearly 1 mm of excess clinical attachment loss. In an older adult population where periodontal support is often already compromised, this cumulative destruction becomes highly clinically relevant, potentially accelerating the timeline to critical attachment loss, tooth mobility, and ultimate tooth loss. Our secondary analyses yielded critical insights into the mechanistic pathways linking complex medical histories to periodontal decline. By categorizing systemic diseases by biological impact rather than utilizing a simple unweighted index, we identified that major cardiovascular conditions are profound independent predictors of clinical attachment loss. Interestingly, immunological conditions such as arthritis and lupus were associated with less severe periodontal destruction in this cohort. We hypothesize this is an incidental, protective effect of the chronic systemic anti-inflammatory regimens (e.g., NSAIDs, DMARDs) central to managing these conditions-a phenomenon uniquely quantified here within a large-scale multimorbidity framework. Secondly, our investigation into specific medication ingredients challenges the assumption that hyposalivation is the sole mechanism correlating polypharmacy to periodontal disease. When isolating active ingredients specifically known to induce severe xerostomia, we did not observe the same magnitude of periodontal destruction as we did when measuring broad polypharmacy (total prescription count). This suggests that 'polypharmacy' serves as a much broader proxy for patient frailty. The total medication burden likely reflects compounding factors such as altered immune competence, generalized physical decline, and a reduced capacity for meticulous oral self-care, rather than functioning exclusively through a single pharmacological pathway like reduced salivary flow. Therefore, while identifying xerostomic drugs remains clinically important, utilizing broad polypharmacy metrics may actually offer dental professionals a more comprehensive indicator of an older adult's overall vulnerability to periodontal progression. A key strength of this research is its use of a large, diverse dataset from electronic health records, which enhances the generalizability of the findings to a real-world clinical population. The dual-analytic approach, using a Random Forest model for prediction and a logistic regression model for inference, is another strength. However, the study has limitations inherent to its retrospective design. The reliance on EHR data means there may be variability in the documentation and a lack of data on unmeasured confounding variables. This study could not control for several critical confounding variables, including specific oral hygiene practices, socioeconomic status, frequency of dental attendance, clinical measurements of salivary flow, detailed functional dependency (frailty) scores, and the specific dosages and durations of the prescribed medications. Moreover, the "black box" nature of the Random Forest model, while powerful for prediction, necessitates the complementary use of regression analysis to explain the underlying relationships. The clinical implications of these findings are significant. The validated predictive model serves as a proof-of-concept for a risk assessment tool that could be integrated into clinical practice to stratify older adults and identify those who may benefit from more intensive preventive care. The results underscore the necessity for dental professionals to conduct thorough medical and medication histories, viewing polypharmacy and multimorbidity not just as background information but as active predictors for periodontal instability (11 , 12). This supports a move towards more integrated care models, where communication between dental and medical providers is essential for managing the oral health of medically complex older adults (4). Future research should aim to prospectively validate this predictive model in a different clinical setting and to investigate the specific biological pathways associating the cumulative burden of disease and medication to periodontal destruction. Studies exploring how changes in medication regimens or the management of systemic conditions correlate with the rate of periodontal progression would also provide invaluable insights. Understanding the specific changes in the oral microbiome associated with polypharmacy in older, dentate individuals represents another important avenue for future investigation (5).

## Conclusions

This study provides robust evidence that both multimorbidity and polypharmacy are significant and clinically relevant, independent predictors for greater periodontal disease severity and more rapid progression in older adults. These findings highlight the necessity for a paradigm shift in geriatric dental care, moving beyond the assessment of individual conditions to consider the cumulative systemic and pharmacological burden. The successful development of a predictive machine learning model underscores the potential for data-driven tools to identify high-risk patients, enabling more targeted preventive strategies. Ultimately, this research calls for greater integration between medical and dental care to effectively manage the complex health needs of the aging population and preserve their oral health and overall well-being.

## Figures and Tables

**Table 1:** Comparison of Geriatric Cohorts Used in Analyses.

Characteristic	Category	Geriatric Cohort (N=5,645)	Geriatric Sub-Cohort (N=2,130)	p-value
Follow-up Time (years)	Mean (SD)	3.5 (3.7)	5.8 (2.9)	<0.001
Age at Baseline (years)	Mean (SD)	72.8 (5.9)	73.1 (5.9)	0.081
Gender				0.482
	Female	3,126 (55.4%)	1,185 (55.6%)	
	Male	2,519 (44.6%)	945 (44.4%)	
Race				0.655
	White	2,398 (42.5%)	903 (42.4%)	
	Black or African American	1,029 (18.2%)	396 (18.6%)	
	Hispanic or Latino (Race)	750 (13.3%)	277 (13.0%)	
	Other / Not Specified	1,468 (26.0%)	554 (26.0%)	
Ethnicity				0.812
	Non-Hispanic	3,061 (54.2%)	1,155 (54.2%)	
	Hispanic	775 (13.7%)	288 (13.5%)	
	Not Specified	1,809 (32.1%)	687 (32.3%)	
Baseline Periodontal Status				
	Mean PD (mm)	3.01 (0.68)	3.02 (0.69)	0.531
	Mean CAL (mm)	2.65 (1.20)	2.67 (1.23)	0.490

1

**Table 2:** Linear Regression Results for Baseline MEAN CAL.

Predictor	Coefficient (mm)	95% Confidence Interval	p-value
Polypharmacy (5-9 meds vs. 0-4)	0.18	(0.11 - 0.25)	<0.001
Hyperpolypharmacy (10+ meds vs. 0-4)	0.29	(0.21 - 0.37)	<0.001
Comorbidity Index (per additional disease)	0.09	(0.06 - 0.12)	<0.001
Smoking Status (Yes vs. No)	0.31	(0.21 - 0.41)	<0.001
Age (per year)	0.02	(0.01 - 0.02)	<0.001
Gender (Male vs. Female)	0.25	(0.19 - 0.31)	<0.001

2

**Table 3:** Linear Regression Results for Baseline MEAN CAL.

Predictor	Coefficient (mm/year)	95% Confidence Interval	p-value
Polypharmacy (5-9 meds vs. 0-4)	0.03	(-0.01 - 0.07)	0.150
Hyperpolypharmacy (10+ meds vs. 0-4)	0.08	(0.03 - 0.13)	0.002
Comorbidity Index (per additional disease)	0.03	(0.01 - 0.05)	0.001
Smoking Status (Yes vs. No)	0.14	(0.06 - 0.22)	<0.001
Age (per year)	0.01	(0.00 - 0.01)	<0.001
Gender (Male vs. Female)	0.05	(0.02 - 0.09)	0.005

3

**Table 4:** Predictive Model Performance on Test Set.

Performance Metric	Value	Interpretation
AUC-ROC	0.81	Good discriminatory ability between Progressor and Stable groups.
Accuracy	89%	Overall percentage of patients correctly classified.
Recall (Sensitivity)	61%	The model successfully identified 61% of all patients who truly progressed.
Precision (PPV)	53%	When the model predicted a patient would progress, it was correct 53% of the time.
Specificity	94%	The model was highly accurate in correctly identifying stable patients.

4

**Table 5:** Secondary Linear Regression Analysis for Baseline MEAN CAL using Biological Disease Clusters and Xerostomic Medication Burden.

Predictor	Coefficient (mm)	95% Confidence Interval	p-value
Disease Clusters			
Major Cardiovascular	0.20	(0.12 – 0.28)	< 0.001
Minor Cardiovascular	0.15	(0.10 – 0.19)	< 0.001
Immunological	-0.11	(-0.16 – -0.06)	< 0.001
Psychiatric / Neurologic	-0.05	(-0.11 – 0.01)	0.074
Metabolic / Endocrine	0.02	(-0.03 – 0.07)	0.494
Pharmacological Burden			
Xerostomic Medication Count (per additional drug)	-0.04	(-0.06 – -0.02)	< 0.001
Covariates			
Smoking Status (Yes vs. No)	0.29	(0.21 – 0.36)	< 0.001
Age (per year)	0.02	(0.01 – 0.02)	< 0.001

The model was adjusted for baseline age, gender, and smoking status. Major Cardiovascular includes history of heart attack, stroke, coronary heart disease, or angina. Minor Cardiovascular includes hypertension, congenital issues, or murmurs. Immunological includes arthritis, lupus, and multiple sclerosis.

## Data Availability

The datasets used and/or analyzed during the current study are available from the corresponding author.
